# Graphic-style stories to engage limited resource communities and promote health: methods for iterative co-design with community representatives

**DOI:** 10.3389/fpubh.2025.1500711

**Published:** 2025-09-08

**Authors:** Susan Gertz, Seung Yeon Lee, Jaqueline Humphries, Luwana Pettus-Ogelsby, Lisa J. Martin, Edith Morris, Keren Mabisi, Eshika Kohli, Kamryn L. Wilson, Carissa Beckham, Vonnie Tawwab, Paula Sherman, Julie Wijesooriya, Shereen Elshaer, Theresa A. Baker, Erin Wagner, Lauren Bates, Susan Hershberger, Melinda Butsch Kovacic

**Affiliations:** ^1^Center for Chemistry Education, Department of Chemistry and Biochemistry, Miami University, Oxford, OH, United States; ^2^Department of Rehabilitation, Exercise, and Nutrition Sciences, College of Allied Health Sciences, University of Cincinnati, Cincinnati, OH, United States; ^3^Cincinnati Children's Hospital Medical Center, Department of Pediatrics, University of Cincinnati College of Medicine, Cincinnati, OH, United States; ^4^West End Community Research Advisory Board, Seven Hills Neighborhood Houses, Cincinnati, OH, United States; ^5^University of Cincinnati Evaluation Services Center, University of Cincinnati, Cincinnati, OH, United States; ^6^Faculty of Medicine, Department of Public Health and Preventive Medicine, Mansoura University, Mansoura, Egypt; ^7^University of Cincinnati Cancer Center, College of Medicine, University of Cincinnati, Cincinnati, OH, United States

**Keywords:** health promotion, co-design, community engagement, graphic-style stories, health literacy, limited resource communities

## Abstract

**Introduction:**

People with lower health literacy and those living in limited-resource communities often experience poorer health outcomes. Leveraging stories for health promotion can be particularly beneficial as stories are more engaging and memorable than other outreach materials. Co-designing health-promotion stories with representatives from target communities ensures their cultural relevance.

**Methods:**

We Engage 4 Health (WE4H), a 20 + member community-academic partnership, developed an iterative co-design and testing process for creating engaging and culturally tailored health promotion stories that initiate meaningful discussions about diverse and often complex health topics. Using a graphic-style format, the stories were designed to be read aloud as the story characters together by program participants and lay educators. Herein, we share three case stories. Surveys and an online focus group gathered feedback from 17 community co-designers from a midwestern US city.

**Results:**

Over six years, WE4H’s flexible co-design cycle facilitated the creation of over 80 stories. Topics included wellness, chronic disease, cancer, citizen science, research participation, and COVID-19/vaccines. Surveys and focus groups indicated that the co-designers felt their ideas were clearly incorporated into the final stories, which made them feel valued and more trusting of the WE4H team. Many developed a sense of ownership of the materials and were more inclined to share the finished products with their communities, strengthening the sustainability of the community-academic partnership and its related outreach programs.

**Discussion:**

WE4H’s community co-design cycle is iterative and highly transferable for creating culturally appropriate health promotion materials on diverse topics for people of varying abilities, backgrounds, and geographies.

## 1 Introduction

Practitioners, policymakers, and researchers recognize the crucial role of community engagement in health promotion and education in the community. Programs that actively involve the community have a greater positive impact on health outcomes compared to those developed without community input (1). Collaborative design and delivery of interventions together by academic researchers and community members have been shown to enhance their effectiveness, while low community engagement in both steps more than often reduces effectiveness (2). Indeed, the process of developing resources along with community members is described using various terms including co-development, co-creation, or co-design. Co-design best describes the process described herein, and is defined as an active collaboration between “experts of their [own] experiences” and researchers, designers, and developers as they “jointly explore and articulate needs and jointly select and implement solutions” (3).

The use of narrative content, such as stories, has previously been shown to have great potential in promoting health within diverse communities (4). Stories have long been successfully used in various health outreach efforts, including human papillomavirus vaccine education and colorectal cancer screening (5, 6). Research in neuroscience and psychology supports the effectiveness of stories in engaging audiences, improving learning, and influencing healthy behaviors (7). Studies using functional magnetic resonance imaging (fMRI) demonstrate that human brains exhibit similar activity when they hear the same story, going beyond language processing to involve high-order brain regions (8, 9). Indeed, others have reported that stories activate parts of the brain in a manner that makes listeners believe that the stories are their own ideas and experiences (10). Stories trigger the synthesis of oxytocin, which enhances empathy and cooperation, as well as the release of dopamine, which keeps listeners engaged and regulates their emotional responses (11, 12). These neurologic mechanisms explain why stories are powerful tools for health promotion and education, making information personally meaningful and improving understanding and recall. Stories can quickly engage participants in community health programs, fostering understanding of health science topics, and enhancing the relevance to their lives. Moreover, stories facilitate community collaboration and easy delivery to and by community members through shared reading (13).

The work described here integrates co-design with the use of stories to promote community health. Unlike stories drafted by health experts, a collaborative approach can be used to allow community members to draw on their personal and collective experiences to promote health in their local communities, therefore ensuring the relevance and significance of the resulting stories. The process of co-designing stories allows co-designers to think differently about relevant events in their lives, leading to greater insight and healing (14). Encouraging readers to imagine their health using stories is particularly beneficial for individuals with limited health literacy and those in medically underserved or marginalized communities who often experience poorer health outcomes and are more likely to favor experiential and global learning approaches over traditional approaches (15, 16). Interventions that are tailored to specific cultural, sex, and age groups are more effective than those that are not tailored (17, 18). Others have shown that interventions that are tailored for and consequently reach subcultural groups of varying abilities from diverse racial and ethnic groups and geographies have the potential to augment health outcomes (19). By incorporating cultural themes and typical conversational language into health promotion stories, their cultural relevance can be increased. Further, the resulting stories may have greater potential to enable intergenerational interactions, which also are more likely to lead to even broader community impact (20).

In this context, the We Engage 4 Health (WE4H) team, a community-academic partnership with 20 + members, developed an iterative story co-design and testing process that has been successfully employed over six years to create over 80 graphic-style stories on various often complex health topics including wellness and chronic diseases, citizen science, health research participation, COVID-19/vaccines, nutrition, cancer risk, and more. The format of these stories uses a comic book or graphic novel style that primarily consists of dialog among characters. The use of graphic-style stories makes it easy for people of all ages and with a wide range of literacy levels to read stories aloud together (a key component of story delivery) because the text is less dense, and images support the text in conveying information (21). Using a graphic-style format, the stories were designed to be read aloud as the characters with community program participants to fuel meaningful discussions and motivate movement along the health behavior change continuum (e.g., from pre-contemplation to contemplation or from preparation to action) (22). Importantly, in an era of short attention spans and competing media, these unique graphic-style stories meet the needs of engaging, memorable, widely understood, actionable, and culturally relevant community health outreach. Indeed, WE4H has utilized community facilitators to share stories to promote health and participation in science and research across numerous community settings, including educational meetings, summer camps, academic clubs, health fairs, citizen science initiatives, and more.

While the use of stories in public health promotion is not a novel concept, WE4H distinguishes itself by its unique approach to utilizing graphic-style stories developed with and for community representatives to help them better understand the topics’ underlying science. Further, rather than being developed by professionals only to be read, the stories can be used by trained lay community educators to initiate meaningful “low-stakes” discussions within their communities. Herein, WE4H’s iterative story co-design process is shared. Included are the community co-designers’ feedback on the process collected via surveys and a focus group, as well as three case stories developed using the process.

## 2 Materials and methods

An iterative and adaptive co-design process was developed that meaningfully leverages the perspectives of representatives of communities of partnering organizations, community organizations, and academic content experts. Study-related activities were evaluated by the Cincinnati Children’s Hospital Medical Center and determined to be exempt from IRB review (IRB# 2019-0659).

### 2.1 Formation of a community-academic partnership

The graphic-style health science and health promotion stories have been created using the co-design and testing cycle within the framework of a community-academic partnership. The main goal of “WE4H” has been to enhance health and science literacy while promoting the health and well-being of individuals residing in underserved communities in the greater Cincinnati area and beyond. The WE4H team is composed of academic faculty, staff, undergraduate and graduate interns, community representatives, and staff members of partnering community organizations. WE4H was planned, developed, and directed by two faculty members – one with expertise in public health and community engagement and the other with expertise in the development of science education materials. Other academic faculty members with content expertise in genetics, nutrition, and environmental health as well as supportive staff members were also invited to join the community-academic partnership at the time of the program’s commencement (which was commensurate with the initiation of grant funding but based on an existing yet narrower partnership).

In addition, high school, undergraduate, and graduate student interns were invited to participate to support the partnership’s efforts. Some of these interns chose to continue their research over several academic years. Core community organization partners of WE4H include staff members at the Seven Hills Neighborhood Houses, a community center in Cincinnati’s West End neighborhood, as well as members of the West End Community Research Advisory Board (WE C-RAB). The institutionally funded WE C-RAB is composed of 16–20 community members at any given time. The group meets monthly at the Seven Hills community center and is responsible for leading health promotion initiatives in the West End and providing insights for research efforts aimed at the West End and similar predominantly underserved minority neighborhoods. The West End neighborhood of Cincinnati had a predominantly African American population (82%) and many of the African Americans in Cincinnati have historical ties to this region. Its median household annual income was approximately $21,000, making it one of the lowest among Cincinnati’s 53 neighborhoods. Only 20% of West End residents over age 25 reported having received formal education beyond high school (23).

Besides core community and academic partners, the WE4H team collaborated with other organizations that commonly catered to urban neighborhoods, particularly those with primarily African American and immigrant residents, to achieve its goal. Many times, these organizations requested stories to support their outreach activities. In addition, the WE4H team relied on other science, education, or medical professionals and faculty who provided input on content accuracy.

Importantly, WE4H leveraged the experiences of their target program participants, most of whom were representatives of communities served by their partnering organizations. Serving as story co-designers, these volunteers provided their personal perspectives on the stories’ topics. For example, many had a disease or health concern relevant to the specific topic of the story or were participants (or guardians of child participants) in local health research studies.

### 2.2 The development of a consistent cast of story characters

**Figure 1:**
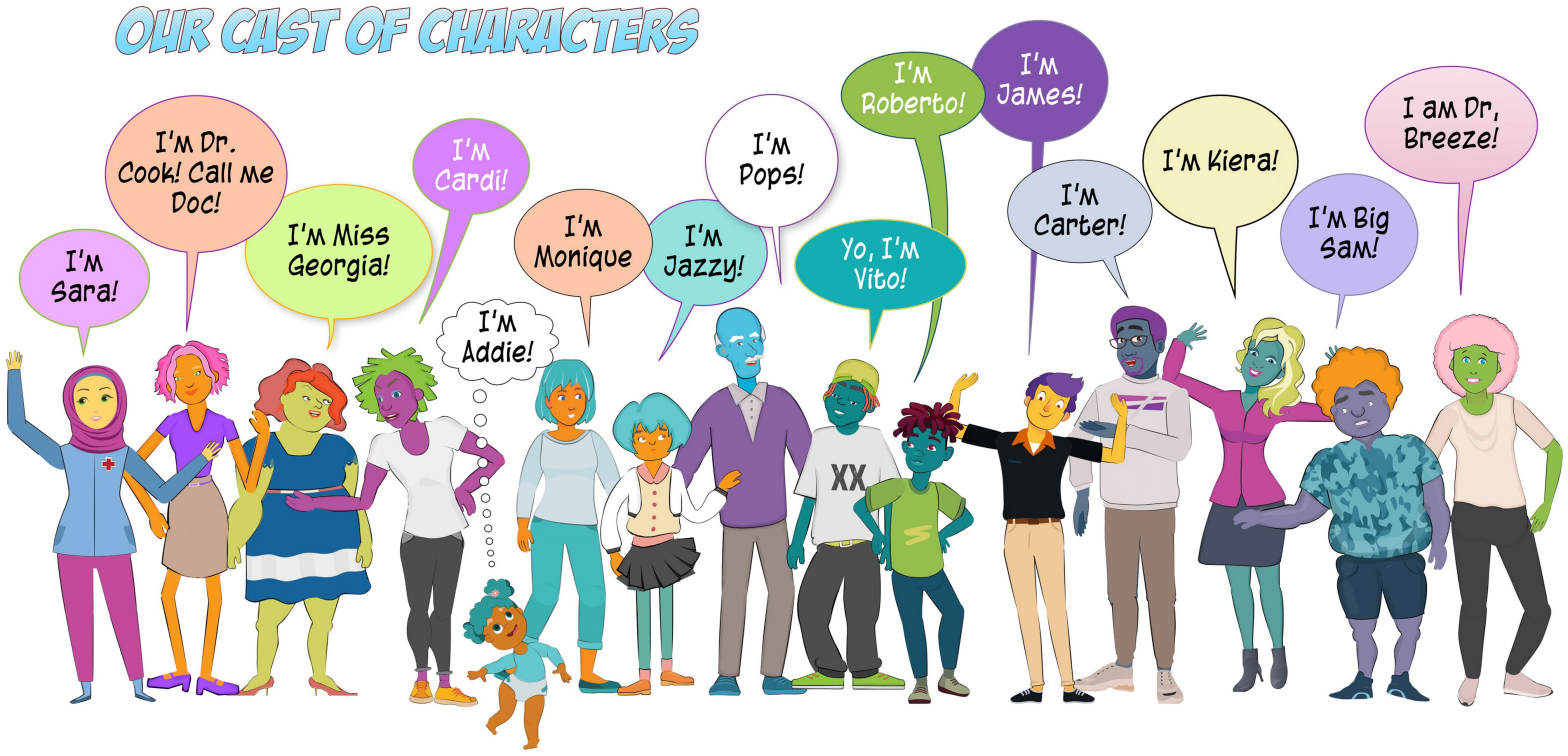
Community co-designed cast of characters for use in community co-designed graphic-style health promotion stories.

The co-design of stories started with developing the characters that consistently appear in WE4H stories with community representatives. The story characters were intentionally designed by community representatives to have unconventional skin and hair features that represent a wide range of tones and textures, without being limited to specific races or ethnicities (Figure 1). This approach ensured that the characters were diverse yet relatable for individuals from all backgrounds. The characters span a broad age range, from children to older adults. In many cases, the characters were modeled on a person or a mix of different people whom the community co-designers knew and respected. As a result, the story characters possess diverse educational and career backgrounds, including physicians, a retired science teacher and cross-country coach, a nutritionist and exercise instructor, a trauma counselor, a researcher, a security guard, and several high school students. They reside in the same community, actively participate in the local community center, and the students attend the same school. Occasionally, the characters’ family and friend relationships are discussed to introduce new circumstances, such as a brother with heart disease, without necessitating the creation of entirely new characters or images, which is a more time intensive process. Importantly, the characters’ background stay consistent between stories allowing program participants or readers to get to “know” the characters over time, thus increasing their overall engagement and identification with the materials.

### 2.3 Selection, training, and roles of co-designers

Story co-designers were often members of the community, but at times were faculty or staff with specific expertise based on the topic of the stories being written. Core community partners from the Seven Hills staff, members of the WE C-RAB or the WE4H community-academic partnership primarily served as co-designers earlier in the project. Later, highly motivated community representatives either volunteered or were invited to participate along the way. More often, health concepts were discussed either during one-on-one or monthly group meetings and meeting attendees volunteered to serve as co-designers. As time went on, new community organizations and faculty/clinical professionals who had been exposed to our materials began asking for new stories that would serve their unique needs. Oftentimes, they volunteered themselves to be co-designers or suggested or provided co-designers if the WE4H team collectively decided to pursue the topic.

At the start, there was no formal training for co-designers. Rather, most co-design efforts were directed by members of the WE4H team. Co-designers were provided a list of question prompts to support the discussion. As the crew of co-designers expanded to help with co-designing completely new stories (in year 2), a comprehensive training was developed that was designed to last one hour though many talkative groups spent 2.5 to 3 hours learning the content over one or more sessions. The training was composed of group story-reading exercises, activities analyzing successful story components, and troubleshooting sample story scripts for natural or conversational speech, inadvertent misconceptions or bias, and inclusion of all characters with knowledge and agency. Co-designers were also provided with a guide detailing each character’s main qualities, such as age, job, interests, and health concerns, along with their backstory, to ensure a consistent portrayal of the characters across multiple stories (see Supplementary Table 1).

Importantly, the training introduced the concepts of a story arc and three big ideas. Indeed, a story arc includes a conflict, an action, and a resolution. All WE4H stories include a conflict or problem that often revolves around the need for information or a need to make a decision. Next, the stories have an action that typically involves the protagonist (a character from our cast) finding a way to obtain the necessary information, skills, and/or support. Each story’s resolution represents the fulfillment of the protagonist’s choices to pursue their goal. The antagonist in WE4H stories is usually a personal or community health challenge, rather than a specific individual.

All WE4H stories also only include three Big Ideas. These Big Ideas are the concepts or principles central to the story. They anchor or connect all the smaller ideas and facts in the story. By limiting each story to only three Big Ideas, the story becomes focused on the most essential ideas that can easily be remembered and shared by readers while forcing the exclusion of non-essential ideas.

For those invited to tailor existing story drafts, a 10-minute video orientation was developed that provided an overview of why stories are used as a key component of WE4H programming, an introduction to the cast of characters, an explanation of the concept of a story, the definition of co-design, and an outline of the tasks and expectations of co-designers (24). While most co-design sessions were held in person at the start of the WE4H partnership, the COVID-19 pandemic led to greater use of virtual co-design meetings as well.

While an ideal co-design process would involve full collaboration with all stakeholder groups at all stages, co-designers often became involved at different points and to varying degrees for each story due to practical constraints such as length of the desired story, co-designer prior experience, deadlines, and authors’ availability. Hence, WE4H’s co-design process has encompassed a continuum ranging from a more consultative approach to a full continuum. For example, the current full-continuum co-design cycle to co-create a 4-panel short health outreach story with approximately 3 to 5 new co-designers from start to finish can now be completed over four one and half hour meetings with staff managing story-related activities and getting co-designer feedback in between meetings.

Regardless of the various degrees of involvement in the process, all co-design members were required to be fully open and responsive to the input provided by other co-designers, and the WE4H team earnestly worked to address concerns or make necessary story changes. At times, this required inviting co-designers to meet and discuss requested changes and to explain the rationale for story wording and/or the inclusion of specific content aligned with evidence-based practices. Further, all co-designers, regardless of the point of initiating collaboration, were offered compensation (a debit or gift card) to thank them for their contributions.

### 2.4 Co-design process and testing cycle of graphic-style stories

**Figure 2:**
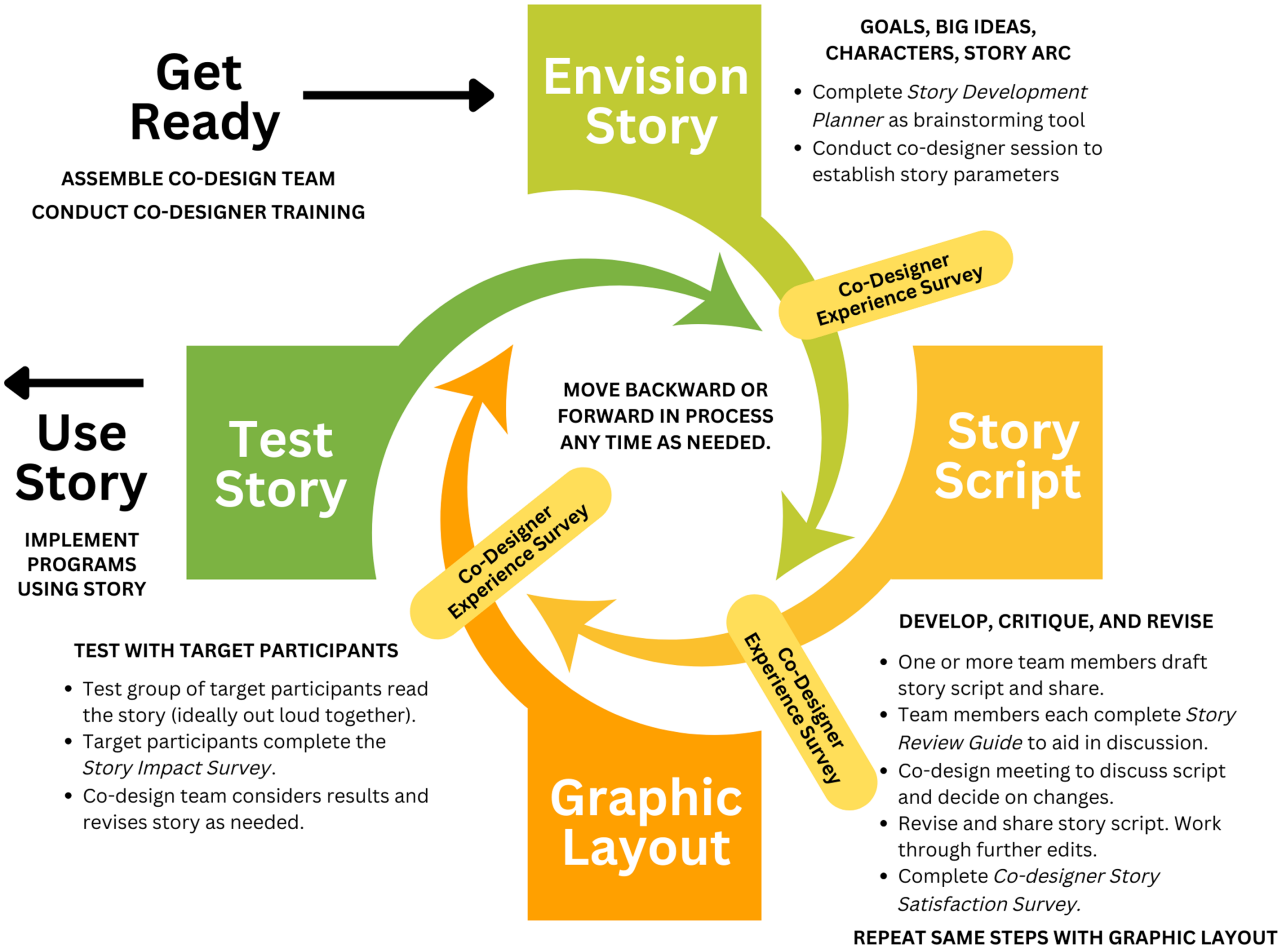
Graphic-style story co-design and testing cycle.

The story co-design and testing cycle diagrammed has evolved and been gradually systematized over six years (Figure 2). It is worth noting that the co-design cycle itself has been co-designed with community members who have been involved in the story-making efforts from the beginning and have played an integral role in determining the most effective process. The story co-design and testing cycle has four major phases: (1) envisioning the story, (2) developing the script, (3) developing the graphic layout, and (4) testing the story. Each of these phases must be completed for every story, although representatives from different co-design groups (WE4H team members, community organizations, target participants, and content experts) often enter or exit the cycle at various points. This flexible cycle allows for maximum involvement of partner organizations and target participants, considering the constraints of time and schedules. Technical steps, such as creating the graphic layout, are typically led by the WE4H staff or intern, and the process may be condensed if necessary to accommodate deadlines. For instance, the WE4H team may take the lead in developing the story concept and script, with co-designers providing iterative feedback on drafts. At each step, revisions were shared with co-designers for approval. If there were disagreements, the co-design team was called to meet to discuss and reach an agreement. Outright disagreements were nearly non-existent, given this approach.

Additionally, as the co-design cycle is an iterative process, any stage can be revisited based on feedback from co-designers. For example, while the “Envision Story” phase establishes the parameters of the story, these parameters may be reevaluated during the “Story Script” phase if the unfolding of the story is not working effectively. To illustrate, the initial plan was for our beloved yet exercise-challenged school security guard, Big Sam, to win a 5-K race in the story arc. However, it did not align with the character’s abilities and would make the story less believable and impactful. In such a case, one of our high school cross-country team members could be involved in helping Big Sam train for a 5-K walk. Alternatively, one of the cross-country runners could be the one training to win a race.

Another iterative process occurs during the “Graphic Layout” phase, which often led to a reassessment of the script. The graphic layout reveals elements of the script that may be challenging or even impossible to discern in a simple text document. For example, if a character speaks for an extended period without interruption, the resulting huge text bubble would take an entire page, making it difficult and unengaging to read. In such cases, different characters may need to be assigned portions of the information to convey (with a plausible reason for their knowledge), or lengthy speeches may need accompanying informational graphics. The placement of page breaks created by the graphic layout can also present challenges, as it may lead to unclear references to content on a different page.

While stories could have been based on the lives of one or more real people, in the end, they were fictitious in nature both to improve information quality and reduce ethical concerns. To ensure the trustworthiness of the iterative process, several strategies were implemented. Specifically, to ensure that feedback from community co-designers on the graphic-style stories was accurately reflected in their development, member checking was conducted by having the revised stories reviewed by the same community members who provided initial input. This was done through prolonged engagement in an iterative process. For each story, most community representatives remained engaged throughout the entire cycle, which contributed to the contextual and cultural relevance of the final products. Although there were some variations in the iterative process across groups, the training was provided prior to the start of the process and during meetings, co-designers were reminded of procedures regularly to ensure dependability - that is consistency. While the graphic-style stories were developed for specific populations—such as African American/Black underserved communities, Hispanic/Latino communities, individuals with autism, and breast cancer survivors—the same iterative process was applied across these diverse groups. This allowed assessment of the transferability of the approach. The successful implementation across these different populations supports the transferability and adaptability of the iterative process. Importantly, at times, when stories were to be used by groups that differed from the original co-design team, a shorter co-design cycle was used and engaged members of the new group to be targeted. For example, eight of the 4-panel cancer stories were originally created by representatives of and developed to target general communities of color at risk for cancer. To improve their relevance to cancer survivors and better highlight their unique risks of second primary cancers, six survivors were asked to review the stories and discuss changes needed to tailor them to the unique needs of their group (details to be included in a different publication).

Through this process, over 80 graphic-style stories have been developed. The resulting stories are unique because of the careful selection and training of co-designers, iterative graphic-style story co-design and testing cycle, and the development of additional materials that complement the stories in various types of community health outreach settings. The resulting stories are therefore ideally suited to be read aloud as the story characters with community members. In doing so, community members quickly understand the material and can engage in often brief but meaningful discussions with trained program volunteers (commonly community members serving as both lay educators and cultural insiders) and staff as they seek to encourage program participants to contemplate adopting healthier behaviors for themselves and for some, to act. The stories are easy for these lay educators to use, and their engagement has the potential to support the sustainability of community outreach programming.

The co-designers were also involved in the development of accompanying hands-on science activities, outreach program guides, program lesson plans, community facilitator checklists as well as several tools to guide the story-making and testing cycle. For example, the Story Development Planner prompts the drafting of required story components while the Story Review Guide asks co-designers to reflect (in advance) on story elements that will be discussed in future co-design meetings. Using the planner, co-designers have a chance to collect their thoughts and maximize the value of the time spent during the meeting. The Co-Designer Experience Survey was developed to be completed by co-designers after each co-design stage. It seeks to identify any instances where co-designers feel underprepared, not heard, or not respected. Repeating this survey through the process allows for corrections to be made. The Co-Designer Story Satisfaction Survey evaluates co-designers’ satisfaction with how well the end product reflected their input. Finally, the Story Impact Survey is given to a test group of target participants reviewing a story to assess its impact. It incorporates a set of questions from the Narrative Quality Assessment Tool (25). This tool was developed and validated for research on culture-centric storytelling for health promotion. It measures three domains of storytelling that are expected to influence attitude and behavior change. Finally, community facilitator checklists are tools to help community members use the stories to facilitate meaningful discussions during specific programs or events and do so with fidelity. While there are some similarities across each stories’ checklist, co-designers were asked to help to decide how the stories are to be introduced, what questions are to be asked, which topics are emphasized, what additional materials would be helpful for the accompanying discussions, and how success was to be measured. These materials and tools are all available for download (26).

### 2.5 Dissemination of stories

Initially, copies of the stories were printed in color via copy machines and directly shared with community members, printed on 18″ x 24″ and 22″ x 28″ foam core boards (https://bluewaveprinting.com/) for use at community health fairs, and displayed via Microsoft PowerPoint during in-person community meetings and virtual meetings. This gave the WE4H team the flexibility to make story updates based on community members’ feedback over time. Later, the stories were organized and printed as high-quality paperback storybooks (Comix Well Spring – a division of Greko Printing and Marketing). With time, the stories were added to the WE4H website individually and, more recently, made available as PDFs on the WE4H website (https://weengage4health.life/) and via the Open Science Framework platform (OSF.io) for download. Instructions on how to print the files are included as well.

### 2.6 Multi methods evaluation of the co-design process

Many co-designers have supported WE4H efforts throughout the project. Some were associated with the West End Community Advisory Board, part of their network, or a WE4H team member’s network. Oftentimes, co-designers were selected because of their roles or familiarity with the topic. For example, co-designers with asthma and/or those parenting a child with asthma were selected for stories on asthma. Similarly, for cancer stories, co-designers were often people who had a previous cancer diagnosis.

The WE4H team contacted 22 previous co-designers who made the most significant contributions via email and phone in September 2022 and invited them to participate in the evaluation. Some co-designers had recently been involved in supporting one or more stories, while others had participated in such endeavors up to three years prior, even before the co-designer training was developed. The evaluation aimed to address three main questions: (1) Do co-designers feel comfortable in the process and that their ideas and time are respected?; (2) Do co-designers feel the completed story reflects their input and meets its objectives for communicating information through a relatable story arc and characters?”; (3) Did the co-design process create a story that the targeted participant understands and relates to? Co-designers were invited to respond to two online surveys namely the Co-Designer Experience Survey and the Co-Designer Story Satisfaction Survey as well as participate in an online focus group held in October 2022.

The purpose of the Co-Designer Experience Survey was to evaluate the experiences of the co-designers while developing the stories with the WE4H team. The survey included eight questions using a 5-point Likert scale with response options including – strongly agree, agree, not sure, disagree, or strongly disagree. The final open-ended question asked the co-designer to share any comments they might have about their experiences.

The purpose of the Co-Designer Story Satisfaction Survey was to evaluate the co-designers’ satisfaction with how well the final end-products reflected their feedback. The survey included four questions using a 5-point Likert scale: Strongly Disagree, Disagree, Not Sure, Agree, and Strongly Agree. The final open-ended question asked the co-designer to share any comments they might have about their stories produced. A $10 gift card was provided to all co-designers completing the surveys. Survey responses (*n* = 17) were summarized using descriptive statistics.

A virtual focus group was conducted by two members of the University of Cincinnati Evaluation Services Center via the Zoom platform. At the start of the focus group, the facilitators explained the purpose of the discussion and that their involvement was both voluntary and an indication of their consent to participate. All co-designers present agreed to participate without hesitation.

The purpose of the focus group was to discuss their perception regarding the process of co-designing health and research-related graphic-style stories for their community. The focus group format was conversational and guided by broad background questions and later centering on the experience of the community co-designer participants. For example, participants started by describing their role as community co-designers, why they decided to become community co-designers, and the preparation and training that they received for the role. The conversation then moved to more specific questions about their personal experience as a co-designer such as describing how their ideas were incorporated, what they would like to see changed in the process, and their feelings of ownership of the product. Finally, they were asked to share thoughts about the stories they were part of developing. The conversation ended by asking the participants if there was anything else that they wanted the academic and evaluation teams to know that we had not asked about. The focus group audio recording and transcript was downloaded from Zoom for the analysis. A $20 gift card was given to participants of the focus group either in person or via US mail.

Two independent qualitative researchers compared the Zoom audio recording and transcription from the focus group (*n* = 5) for accuracy, and coded and analyzed them using thematic analysis (27) in MAXQDA (28). The findings were triangulated with the survey data, giving them additional meaning and summary. Thereafter, WE4H team members also affirmed the findings.

## 3 Results

### 3.1 Online co-designer surveys

In total, 17 co-designers completed the online surveys and 5 participated in the online focus group (Table 1). Of the 17 survey respondents, 41.2% were community members. More than half reported being Black/African American. All but one were female. Most of these women had been co-designers for multiple stories. Only women aged 60 or up were available for the focus group.

**Table 1:** Participating co-designer characteristics.

Characteristics	Survey respondents*	Focus group participants
	(*n* = 17)	(*n* = 5)
Age (%)		
19–29	4	0
30–39	1	0
40–49	3	0
50–59	3	0
60 and above	6	5 (100)
Female (%)	16	5
Male	1	0
Race/ethnicity (%)		
Black/African American	9 (52.9)	5 (100)
White/Caucasian	6 (35.2)	0
Asian	1 (5.8)	0
Asian/White	1 (5.8)	0
Education level (%)		
Less than high school	2 (11.7)	0
High school	1 (5.8)	1 (20)
Vocational /some college	5 (29.4)	2 (40)
College graduate	9 (52.9)	2 (40)
Co-designer category		
WE4H staff	2 (11.7)	1 (20)
WE4H intern	4 (23.5)	0
Content expert	4 (23.5)	0
Community member	7 (41.1)	4 (80)

*Includes focus group participants.

**Figure 3:**
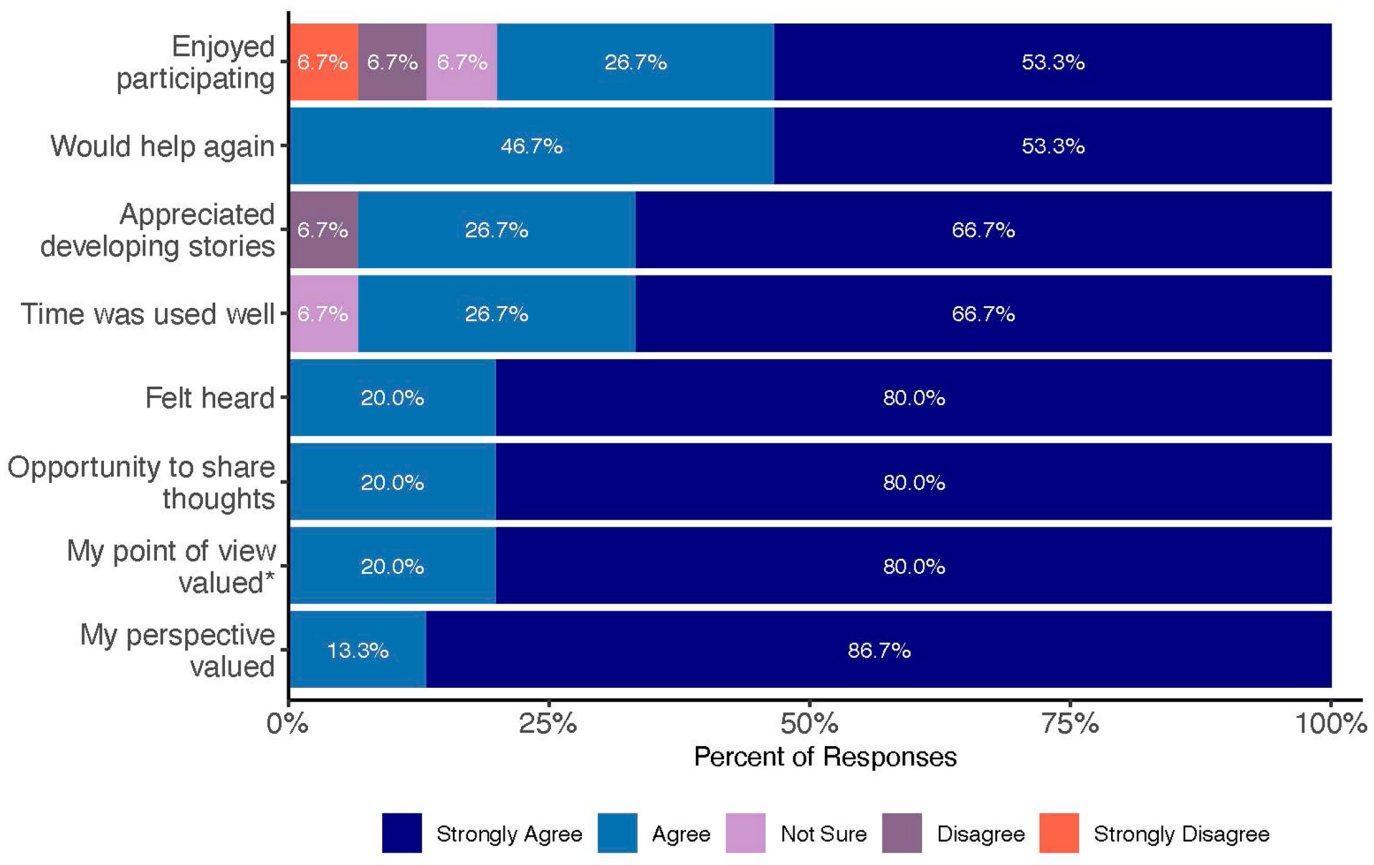
Co-designer experience survey results (*N* = 17).

The Co-Designer Experience survey indicated that between 93 and 100 percent of survey respondents agreed or strongly agreed with positive statements about their experience (Figure 3). Indeed, one co-designer commented on her survey that she “loved the experience,” while another said that “it felt good to be really heard, understood, and appreciated.” A third co-designer felt that it was a great learning experience and that she learned a lot about community health and the many ways people interpret information. She, too, appreciated the passion the other co-designers had for the work. In response to “My point of view was valued,” a few co-designers responded, “not sure,” “disagree,” or “strongly disagree.” However, these responses were from co-designers who only supported the project early on. Indeed, the co-designer training and iterative cycle were developed directly in response to some early challenges with the goal of providing co-designers with clear directions and encouraging them to share their opinions. As a result, survey responses from those who participated later in the project were all favorable.

The Story Satisfaction survey results showed that 95% of respondents strongly agreed that their input was incorporated into the final story, and 100% of respondents strongly agreed that they were valued as co-designers and would participate as co-designers again. Furthermore, survey respondents indicated that they “felt proud” that they were able to contribute to the story and that seeing the final product was “rewarding.” One said, that “It was really tough at first, but after the countless edits and changes, I feel like the stories I helped make were good and had a lot of potential to educate people but also find enjoyment in the stories as well.”

### 3.2 Online co-designer focus group

Among 17 survey respondents, four community co-designers and one staff member (originally a community member) also participated in a focus group. Two of the participants had been active in co-designing the stories since WE4H’s beginning and, therefore, had not received any formal training to be a co-designer *per se,* while the others had been invited to support story co-design as time went on and may have received some elements of what later became our formal training program for co-designers. The formal training was developed to make it easier to more efficiently on-board co-designers. When specifically asked about their training, most stated that it had been informal. As one participant said.

*“I didn’t even realize I was really a co-designer. I was just We Engaged for Health was there & they said they this is what we wanna do, what do you think and they just start bringing this to us and we just started giving our opinion. I didn’t even know I was really doing anything but helping write a story.”*

Throughout the discussion, they were positive about the co-design process. They noted that in the process of developing the stories, their academic partners would iteratively call them to ask about their reaction to the stories and the story graphics. Not only did academic partners ask them what they thought, but the academic partners listened to their suggestions, and they could see their suggestions implemented in the stories. One participant described it this way:

*“I enjoyed actually just giving the input and having the confidence in knowing that my input would be taken seriously and somehow incorporated into the story so right now it’s got me feeling like I’m a thang.”*

Being heard allowed them to take ownership of the stories and was exemplified in the enthusiasm with which they took these stories into the community. Several mentioned the feeling of pride they had as they shared the stories with other community members. Additionally, their ownership of the stories sustained their comfort with using the story booklets at events in their churches, community centers, and other formal and informal meeting places. They mentioned that community members easily engaged with them around the stories even asking how they could become a part of the program. Some participants acknowledged that the academic partners worked diligently to include both a scientific basis in the stories as well as their community’s culture which further helped them to feel like valued members of the WE4H community-academic partnership.

Many were invited because they themselves or someone in their family had experienced the illness or disease topic of the story (for example, people with children with asthma informed the pediatric-focused asthma stories, etc.). These co-designers were able to share their voices and perspectives and learn from the process.

A thematic analysis of the focus group discussions identified three main themes. Themes emerging from the data were (1) *Authentic community-academic partnerships created course content*, (2) *Target community groups were responsive to content created by community peers, and (3) Community co-designers were interested in learning about common community health problems.* Overall, the results provide evidence of authentically engaged community co-designers. Each of the themes are discussed in more depth below.

#### 3.2.1 Theme 1: Authentic community-academic partnerships created course content

The training offered to community co-designers fostered the partnership between the academic team and the community co-designers laying a foundation for the community to “own” the program and become leaders in improving health and research literacy in their communities. A system of mutual dialog and respect gave the community co-designers confidence to share their innate knowledge of the community. One of the focus group participants described the mutuality by giving an example:

*“Yeah, they just asked us to be honest with our opinion. Just give our honest opinion on whatever they were asking us & we did. And what I loved about it was they listened to us at every point they listened to us. They listened to us because when it came out in the story you can see that they listened to us because it was in writing. They valued my opinion in every way.”*

Understanding the culture and speaking the language of the community coupled with the science of the academic partners, offered community members a means to improve health and research literacy in a “user-friendly” manner. One participant said.

*“…because in the arena that the professionals are in they truly don’t understand that the community at large is not going to understand certain words that they use and the role that we played was just telling them that ain’t gonna work, there’s another word for that that the community will understand because that is how you are going to reach the community. That plays into meeting them where they are understand that they don’t know what you know.”*

The openness of the academic partners to hear community members’ opinions was an important component of the successful story co-design. The focus group participants repeatedly mentioned the respect and sense of equality in working with academic partners at all levels (PI, staff, student intern). This coupled with the evidence that their recommendations were apparent in the written stories was further evidence that they had been heard. One participant shared how this had made her feel: *“You know and coming from a community of color, to finally be in a position where you know for a fact that you are being listened to is wonderful.”*

Showing the co-design training video at the end of the focus group further substantiated the mutual respect between the academic and community partners. When asked for their thoughts on the training video, the community co-designers indicated how they appreciated that the video made it clear that the stories were being designed to be read aloud by program participants and that the stories’ focus was to create opportunities for meaningful discussion about the stories’ big ideas. The participants were also supportive of the training video’s attempt to create greater structure in helping community representatives like them to become community co-designers. One participant said.

*“I think this is a very clear informational board - it kinda gives you a feel about what you will be doing and what’s expected of you. It’s kind of exciting too, it answers some of your curiosity, so you’ll know this is what I’m going to be doing this is what they expect so I liked the board a lot.”*

Participants were also free to make recommendations for the video. The presenter affirmed each recommendation while noting needed changes. She stated: *“That is a really good idea. We can do that.”* One participant recommended utilizing animation in the presentation to better keep the audience’s attention. They also further offered to have a family member provide “voiceovers” for the animation. Her recommendation was warmly received expressing a desire to continue the conversation as the video moves to completion.

The focus group discussion provided evidence of the strong WE4H community-academic partnership that had been developed in part by partnering with co-designers. This partnership and the resulting ownership within the community laid the groundwork for an opportunity to impact health and research literacy in the community.

#### 3.2.2 Theme 2: Target community groups were responsive to content created by community peers

The focus group participants shared their belief that communities receive information better when it is presented by people who “look like them,” know the language of the community, and understand the culture of the community. Gaining acceptance is critically important for engaging a community and this is best done through speaking the language and having an insider’s understanding of the culture from those representatives of the community. Community “insiders” are trusted and offer reliable information.

*“Well, I believe that in order for one to reach the community, my community of color, they need to see people of color and they need to be able to relate to the individuals that they see and I know they see that within me.”*

The community co-designers saw themselves as a liaison between the academic world, the world of technical science language and the language of the community. They took the words of the scientists and put it into the language of the community so that it would be better understood. A participant explained this intermediary process:

*“Have academia break down certain, and I can say that because in the arena that the professionals are in they truly don’t understand that the community at large is not going to understand certain words that they use and the role that we played was just telling them that ain’t gonna work there’s another word for that that the community will understand because that is how you are going to reach the community. That plays into meeting them where they are; understand that they don’t know what you know.”*

The participant went on to say that when they first read the stories, the discrepancy between the academic language and the language of the community was noticeable. She believed that this would lead to misunderstanding, fear, and a general non-acceptance of the information and asked for the language to be changed. This exemplified the level of respect and equality that existed between the academic project team and the community co-designers. Active listening was an important concept in making WE4H successful.

#### 3.2.3 Theme 3: Community co-designers were interested in learning about common community health problems

The focus group participants were all community co-designers who were avid learners. They shared that they enjoyed learning about how best to promote health in their communities, taking pride in the health of their community, and were eager to share the information with their communities. Additionally, they wanted to understand some basic information about health research so that they could speak knowledgeably with their health care providers and encourage community members to also engage with their providers and with researchers. Taking pride in this work was mentioned multiple times during the discussion.

Some of the participants reported that they were interested in being involved because they had family members with health issues, and they could learn more about them as well as assist in making the story understandable in the community. As one participant said:

*“I read the story regarding the eczema and asthma story and that’s pretty much how I got involved because my children and my grandchildren have dealt with eczema and asthma and I was really trying to find out more about what I could do, what I could share with them that they could do to make their healthcare better and even today after rereading the series I saw something that I didn’t see before and it made me want to change something else for their health.”*

The participants went on to say how proud they felt to bring this information to their community and to bring it in a way that was backed by science. Community co-designers who had been supporting WE4H the longest were most comfortable with taking the information out into their communities independently. They noted that if they were asked questions that they could not answer they knew that they had back-up from the academic partners and hence were comfortable in saying they did not know but would find out the answer. While newer members of the group had done less community teaching, they hoped to gain confidence to soon become more independent. The motivation for sharing the information in the community was readily apparent in the focus group discussion. One participant enthusiastically said.

*“I am so proud that every week we had a health fair I told [academic team member] I wanna do HPV, we wanna do skincare, we wanna do the hand washing, because it was just so important for us to get that information out. We were ready to share it. I mean we was going out every weekend we were having a ball.”*

The community co-designers used story content and hands-on experiments to support the content in talking with community members about health. Using science-backed information in community presentations was essential to providing good health information to people.

*“I’m not sure if I said this or not is what I love about We Engage 4 Health is when we’re out there showing them the science behind what we’re saying and it makes a difference cause we’ve been told all our life to eat fresh fruits and vegetables we’re able to show them the science behind the importance of fresh fruits and vegetables because we show them with the iodine, we showing them, and it is powerful.”*

The focus group discussion provided strong evidence of the usefulness of co-design in producing high quality health promotion materials that were respectful of the community’s culture and language. Further our results show how co-design positively enables meaningful partnership between community and academic partners – particularly those that may have differing life experiences and cultural backgrounds.

### 3.3 Case studies of resulting stories

Using the co-design process described in the Materials and Methods WE4H co-created over 80 unique health outreach graphic-style stories with some distinguishing features (Table 2). Importantly, the stories cover a variety of topics in varied formats and settings (Table 3).

**Table 2:** Unique features of WE4H graphic-style stories.

Consistent cast of characters	A cast of community co-designed characters appears throughout the stories. Program participants get to “know” them over the programming, increasing their engagement and identification.
Community co-design	Stories are co-designed by members of the program’s target communities to ensure they are locally and culturally relevant.
Graphic-style stories	The use of graphic style stories makes it easy for people of all ages and with a wide range of literacy levels to read stories aloud together.
3 big ideas	The stories are written to focus on only three big ideas making these ideas memorable and shareable.
Easy for lay educators to use	The stories are easy for community lay educators or cultural insiders to use in a variety of settings to spur meaningful discussions. This supports program sustainability.

**Table 3:** Story series and their intended uses and topics.

**Health is Happenin’ RAP** Twelve stories (10–20 short comic panels each) designed to be offered across 6–12 sessions. Intended use: Used in a series of sessions incorporating stories plus hands-on activities. Topics include wellness plus decreasing risk of and helping to manage chronic diseases such as diabetes, high blood pressure, and asthma with healthy lifestyle choices.
**Citizen Science RAP** Twelve stories (20–25 short comic panels each) designed to be offered across 6–12 sessions. Intended use: in a series of sessions incorporating stories plus hands-on activities. Topics include understanding what citizen science is and how citizen scientists can design experiments, collect data, and analyze and report on data.
**Health fair panels** 35 + stories each with 3 graphic-style panels and a 4^th^ Challenges and Actions panel. Intended use: for displaying on tables and read out loud by health fair attendees as they visit each table. Topics include understanding health risk, learning about cancer cells, protective and risk factors, various types of cancer (breast, colon, lung, skin, prostate, and head/neck/cervical), the importance of primary care providers, etc.
**COVID-19 and vaccine education** Three stories in book form (30–45 panels each) plus a set of ten 1-panel graphic FAQs. Intended use: as public/website resources plus outreach by trained community Health Champions. Topics include how vaccines work, development of COVID-19 vaccines, common objections to vaccines.
**Human subjects research education** Two 20–25 panel stories in book form designed to initiate longer discussions and two 5-panel stories for brief discussions. Intended use: Outreach by cultural insiders trained as Community Research Advocates to discuss and help the public understand human subjects research and the value of being involved in it (aka increase “research readiness”). Topics include the purpose of research, the need for people of all ages/backgrounds to participate so the results are relevant for all, and the rules and regulations that ensure the safety of participants. A Review Guide supports decision making.
**Research study participation education** One 30-panel story in book form and one 12-panel shorter version. Intended use: Educating research study participants about the purposes and procedures for the specific study they are enrolled in, encouraging study participants to complete their annual study visit over the life of the five year study. Topics include the purpose of the study, the types of data collected in the study, how study participants benefit directly, and how children may benefit in the future from the knowledge gained by the study.
**Eyewitness Community Survey (ECS) education** Three 40–50 panel stories in both book and video formats Intended use: Providing training for citizen scientists to collect local environmental data. Topics include consent, the impact of the environment on health, and instructions on using the ECS tool to make and record observations in their own communities.

Herein, are three case studies to illustrate the applicability of the co-design process with various community groups. While these processes resulted in effective end-products, many lessons were learned along the way that allowed the WE4H team to optimize the process and create a better collaborative effort. Indeed, these case studies illustrate the ability of the co-design process to self-correct along the way if everyone involved is open to its iterative nature. Many of the lessons learned resulted in developing the co-designer training, surveys, and other tools discussed previously. These did not exist at the start of the project and gradually became available to ensure we were meeting our goals going forward.

#### 3.3.1 Case study 1: Nutrition health fair panels developed with community matters

Community Matters, a social service agency in Cincinnati’s Lower Price Hill neighborhood, contacted We Engage 4 Health regarding participation in a health fair with the theme of healthy food and urban gardening. At the time, WE4H had developed a series of stories for their Health is Happenin’ RAP program (RAP stood for Recognize, Ask, and Promote), several of which touched upon the role of food in minimizing chronic disease and staying healthier and fit the theme of the health fair. The Health is Happenin’ RAP stories were developed for a program where people attended sessions about two hours long, reading two stories and doing two hands-on activities. As such, the Health is Happenin’ RAP stories were much too long for the type of “walk by” interactions typical of health fairs. Through co-design discussions, a new concept emerged: extremely short, three-panel health fair stories to be placed on tables as large posters and engage visitors in reading aloud for about 3–5 minute. Four topics from Health is Happenin’ RAP with a nutrition connection were developed into a set of health fair stories: lead exposure, air quality, and asthma, stress, and antioxidants in food. A fourth panel summarizing health challenges and healthy actions was added. While COVID-19 restrictions led to the cancellation of the initial event, the health fair story panel concept was extended to the development of 26 + health fair stories (and counting) covering topics including reducing health risks, cancer prevention and screening, the role of primary care practitioners, and more. These mini stories have been successfully used in numerous health fairs, where nearly 100% of attendees said they would enjoy learning about new topics in the same way. More recently, they have begun to be used during small community group meetings to invite dialog about targeted health issues being experienced by those groups. A compilation of the current health fairs entitled “Engaging Conversations for Community Health” can be freely downloaded (29).

#### 3.3.2 Case study 2: Co-creation of “Research Ready” with the WE C-RAB

The West End Community Research Advisory Board (WE C-RAB) is an institutionally supported board that has been working together since 2017 to provide a community perspective to health researchers from Cincinnati Children’s Hospital Medical Center and the University of Cincinnati as well as support health promotion efforts in the West End. In 2020, members of WE C-RAB expressed the desire to “be able to better talk to their family, friends, and neighbors about research and its potential impact on their health.” As some WE4H team members also have a connection to WE C-RAB, the board was aware of the graphic stories, and the possibility of such a story to fill this need was proposed. WE C-RAB members are knowledgeable about the important measures that safeguard research participants and the important role of diverse communities in participating. They wanted the story to express these ideas. Unlike most of the other WE4H stories, story panels for what is now called “Research Ready” initially were brought together from various available WE4H stories and shared with both WE C-RAB and WE4H team members. Refinements thereafter made by the WE4H team were brought to the monthly WE C-RAB meetings for review and discussion. As “experts of their experiences,” WE C-RAB members identified issues like overly difficult vocabulary and too-dense information that would cause the story to be less effective. WE C-RAB’s community coordinator, also a member of the Cincinnati Children’s Hospital Medical Center Institutional Review Board (IRB) as well as researchers also reviewed the story to ensure that the key ethics concepts were accurately represented while being simplified.

In the story, a teen named Vito is invited by his family doctor to be in a cancer risk factor study because of a family history of cancer. Vito’s not sure what to do and asks for advice from Pops, a retired science teacher, and Carter, a researcher at a local university. Vito knows them from the community center. Vito learns about becoming “research ready,” which is defined as understanding the 3 P’s: the *purpose* of health research, how participants are *protected* from harm when participating, and why it is important that people from all backgrounds and walks of life *participate*. The story ends with Vito saying he’ll have to think about it.

A version of the story was deemed by all co-creators to be good enough to share with WE C-RAB members to try out with their family, friends, and neighbors. To provide more structure to their outreach, a program called “Research Ready” was developed and included facilitator training for both WE C-RAB members and interested community members. The outreach plan included an activity where participants looked at sample clinical research studies and discussed whether they would want to participate. Participants enjoyed the story but seemed to struggle to do the activity. In retrospect, the co-design team realized that the story never modeled the desired behavior of reading the research announcement to answer questions and decide about participation in a study. Since the story did not model the behavior, participants did not have the chance to “*mimic behaviors that they have seen modeled*.” The behavior was “*recommended but not demonstrated*” (30). Therefore, a Research Study Review Guide was added that included a list of questions to either ask themselves or ask study staff to inform their decisions.

True to the iterative nature of the story development process, despite having moved so far in development, the story was further revamped. Now, after learning the 3 P’s, Pops prompts Vito with a series of questions to consider, and Vito can answer them by reading the research announcement his doctor gave him. These questions were also included in a Research Study Review Guide. The final version of the story (now with 20 panels) and guide can be freely downloaded (31).

**Figure 4:**
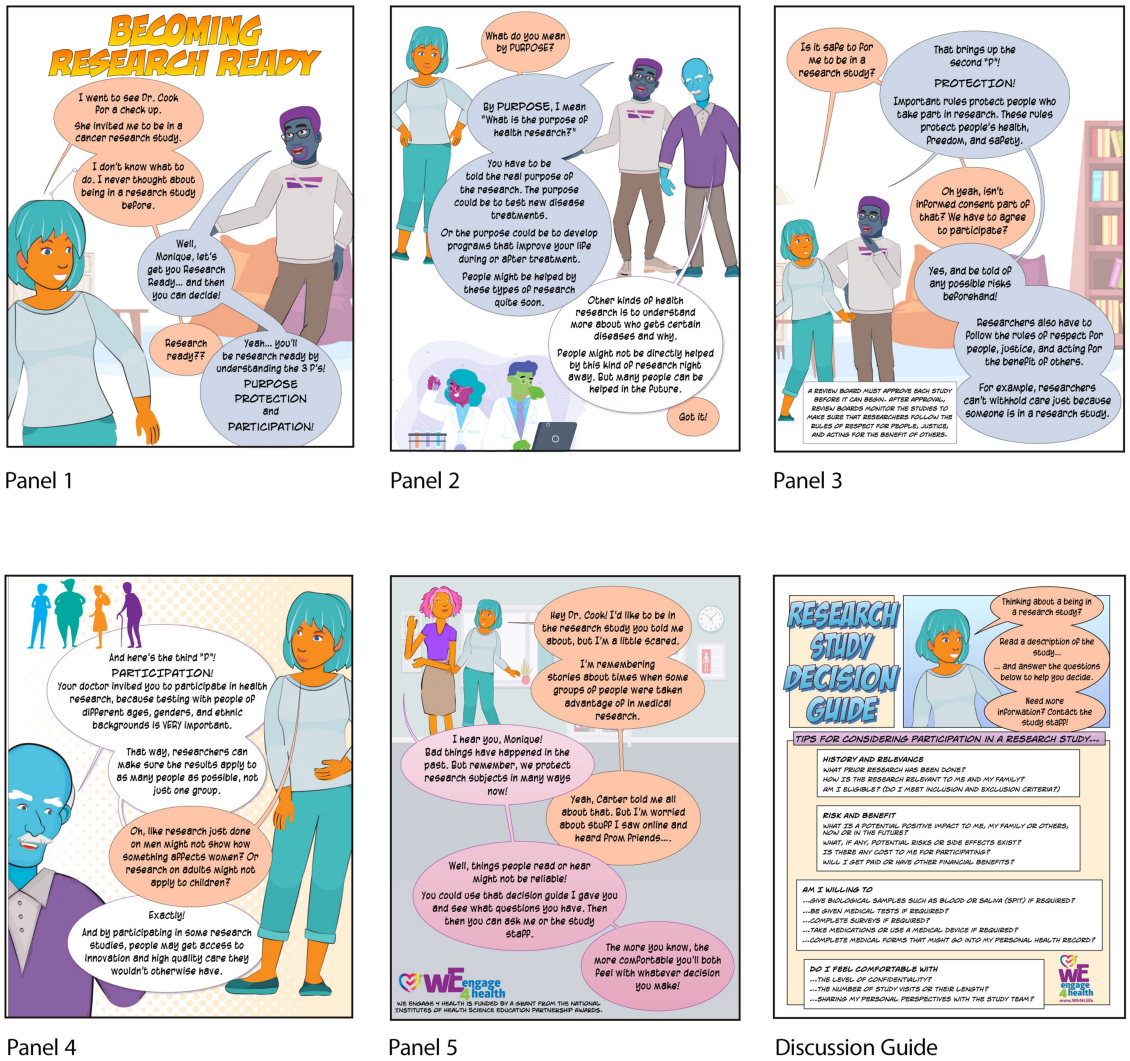
Short Becoming Research Ready story to introduce health fair/event attendees to the concept of research readiness and encourage them to take the longer storybook home to read and discuss research participation with their families. Included also is the accompanying Research Study Review Guide that they can use to help them personaly decide about participating in specific research studies in the future.

The iterative nature of story development continued with the identified need for a very short summary of the story in five panels to introduce visitors to the idea of being “Research Ready” at community health fairs and invite them to take a copy of the full-length story to share with their families and friends. More recently, similar stories entitled “Becoming Research Ready” were co-created, mirroring the first with a group of six cancer survivors to encourage patients with cancer to consider participation in research, including cancer-related clinical trials. While the story’s main character is Monique (a breast cancer survivor) rather than Vito, both are designed similarly to the original with Monique trying to decide about whether she wants to participate in a cancer-related research study or not. The five-panel story is designed for use at cancer-related events (Figure 4).

#### 3.3.3 Case study 3: Co-creation of “Learning about MPAACH” and “following the allergic march” with MPAACH study research participants

Researchers at Cincinnati Children’s Hospital Medical Center contacted WE4H regarding their interest in using a graphic-style story to promote research subject participation and adherence over the 5 years of their research study. The study, titled Mechanisms of Progression of Atopic Dermatitis to Asthma in Children (MPAACH), enrolled children three years old or younger and followed for 5 years to learn about the progression of eczema to asthma. A WE4H intern was assigned to meet with MPAACH clinical research coordinators to learn about the research study and to shadow MPAACH year 4 research visits. The intern discovered that many families canceled at the last minute or were “no-shows.” Further, during visits, she found that the allergy skin prick tests and blood draws were very stressful for children and caregivers, and caregivers frequently asked many questions about the purpose of these tests. While these tests had been discussed during the consent process 4 years prior, caregivers of MPAACH participants needed reminders. Based on these conversations and observations, the intern drafted a short script for a story to introduce potential new participants and a longer story to support the consent of newly enrolled participants or to remind existing participants about the purpose of the MPAACH study and its accompanying tests. Each story included three big ideas which included: (1) MPAACH studies the allergic march (where babies with eczema are more likely to have asthma later in life), (2) each year at the MPAACH visit scientists collect biological samples and complete surveys and tests, and attending all five years of visits is important to understanding the allergic march, and (3) participants benefit directly by receiving allergy test results for their own children and other children will benefit in the future from the knowledge gained. After several iterative rounds of refinement with WE4H staff, both scripts were placed in the graphic-style format using Comic Life software. The resulting shorter story was 12 comic panels. The longer story was 30 panels.

Both stories were shared with the caregivers of three MPAACH children who had completed 4 of the 5 years of the study. These co-designers participated in co-design orientation and training and then provided feedback. They identified terminology and other language in the story that was confusing to them personally or that they deemed potentially confusing to other MPAACH participants. One co-designer felt very strongly that the stories were not relatable to caregivers of young children because although a baby was referred to, s/he was never shown as a character.

Another co-designer felt strongly that the character “Pops,” a retired science teacher, looked “old and creepy,” and would not be seen as a reliable source of information. The group also struggled with the term “atopic march” and instead indicated the word “allergic march” was easier to understand. Along with their input for improvement, the co-designers emphasized how much they had learned from the story, especially about the purpose of the MPAACH study and the reason for each test provided, which they feel they previously never really understood despite the review offered at consent. They preferred the longer story to the shorter one because it provided a much greater level of detail. Based on this feedback, clarity and organization were improved, a baby character “Addie” was added, and Pops was redesigned. The MPAACH research team sent existing study participants a link to view the stories online. Others were offered a flyer with a QR code linking to both stories during their study visits (32).

## 4 Discussion

An iterative and adaptive co-design process has been developed that meaningfully leverages the perspectives of representatives of partnering communities, community organizations, and academic content experts to develop graphic-style stories for health promotion in underserved communities. This co-design process has been applied to the development of over 80 stories on diverse topics, which are used with varying audiences and in numerous contexts. The study was conducted using an integrated framework that reflects multiple philosophical approaches of ontology, epistemology, and axiology (33). Story creation was carried out through an iterative, co-design process involving community members and academic partners. This participatory process embodies a contextual and experiential epistemology, grounded in the lived experiences of participants and shaped by culturally relevant and socially constructed meanings, hallmarks of both social constructivist and experiential epistemological perspectives. The study’s axiological stance is rooted in values of inclusivity, empowerment, equity, and respect for community voices. Through iterative engagement, it prioritizes trust-building, cultural relevance, and shared ownership of graphic-style stories. This value-driven approach aims to address health disparities by collaboratively developing health promotion materials that resonate with the communities they serve.

Early co-designed story sets focused on improving community members’ overall wellness and reducing the risk of chronic disease (the Health is Happenin’ RAP series of stories and program) and becoming involved in health research as citizen scientists (Citizen Science RAP and Eyewitness Community Survey series of stories and programs). Thereafter, new stories and programs emerged organically. As community and academic partners observed the usefulness of the stories in events and programs, they were inspired to share their own story ideas with the WE4H team. This sharing resulted in the expansion of the co-design crew and the development of new outreach stories and materials, such as for recruiting diverse participants for human health research, educating families of child research subjects, informing citizens about the health impacts of water quality, and promoting childhood vaccinations and cancer screenings in underserved populations. Similarly, high school and undergraduate interns involved in our programs also created stories on health challenges their own families had experienced, including autism and Alzheimer’s disease. Others created a set of 12 stories to educate elementary and middle school runners about the sport of running. “Running is Fun” includes detailed instructions for coaches and parents on how to use the materials and Knowledge Checks after the stories emphasize the stories’ main points.

The delivery of the stories involves communal reading where participants assume the roles of the characters and thereafter engage in meaningful discussions and inquiry activities. This communal reading and discussion experience, termed “story sharing,” create a level playing field for participants, stimulates similar brain activity in readers and listeners, and facilitates two-way interactions between the facilitator and all participants (34). People of all ages and backgrounds are eager to participate as readers, and any challenges with unfamiliar words are overcome with support from the group. The stories’ unique characters and settings seem to disinhibit participants, allowing them to engage in discussions and reflect on the characters’ concerns while learning valuable information for themselves.

The format and length of the stories vary to adapt to different settings and time constraints. For example, in a health fair setting, stories are printed on large poster boards that stand on tables, and individuals are invited to read them aloud together. In clinic offices and home health care settings where time is at a premium, quick one-panel stories addressing specific concerns can be read aloud in minutes. Longer programs feature storybooks with detailed descriptions of the underlying science of specific health challenges. (e.g., our Take Your Best Shot, Vaccine Victory, and Voices of Vaccine books).

Developing the stories is not a simple task, and involving community co-designers adds time, communication, and facilitation requirements to the process (35). Some professionals may perceive this as an unnecessary burden, expecting a quicker development and implementation process without external input (35). It is crucial to educate both community co-designers and health/academic professionals about the value and goals of the co-design process to foster a collaborative environment where the stakeholders’ input is respected (36). It involves giving community representatives a strong voice and treating their experience-based knowledge as equally important and relevant as research-based knowledge (35).

The survey data and focus group analysis of the community co-design experience demonstrate that the iterative story co-design cycle effectively incorporates community perspectives and creates a sense of pride and ownership among co-designers. In contrast, Lorini, et al. (37), reported that their community co-designers had lower self-efficacy in their ability to make meaningful contributions after participating in the process. WE4H co-designers, whether informally trained through experience or through co-designer training, felt well-prepared to support the process and appreciate the iterative nature of the co-design process. The resulting stories are particularly effective in communicating complex information and stimulating meaningful discussions among their peers. Indeed, tailored materials have been shown to better encourage agency and behavior change (38).

Although the iterative process is valuable for developing culturally and contextually relevant materials that reflect the voices of community members, it has several possible limitations. The story development cycle requires community partnerships which may require a significant investment in building trust and rapport to ensure their meaningful contributions. At the start, these partnerships can be time-consuming for both academic and community partners. Without existing community partnerships or prior community collaborations, this approach may take longer to implement compared to traditionally prepared health promotion and educational materials. Some community co-designers may find participating burdensome. Indeed, the length of community co-designers’ required commitment varied depending on the types of stories being developed. For example, community co-designers who helped to develop shorter 4-panel stories that began at the Envision Story phase on average attended 4 meetings of 1.5 h each as well as completed surveys outside of the cycle. The commitment was much less if co-designers began at the Story Script or Graphic Layout Phase or were given a completed story draft to tailor together. The commitment of those supporting the development of longer stories was much greater. Still, it was common for story co-designers to be eager to support the development of more than one story, suggesting the process was not burdensome for most. Challenges experienced by co-designers of longer stories were often offset by the value of the process and its outcomes, the creation of educational materials that are relevant and meaningful to their communities.

Given the strong influence of the co-designers, the resulting stories developed through this approach tended to be specific to the cultural context or health condition being addressed, such as African American/Black breast cancer survivors or individuals with autism. Nevertheless, the iterative process is adaptable and can be tailored to other groups. It has been successfully applied across diverse populations and settings, demonstrating its transferability. While at times the process can be lengthy, its depth and inclusivity contribute to the development of impactful and community-centered materials.

No matter how inclusive, participatory, or democratic the process is, the institutions doing the research and design often benefit more than the communities do (39). Compensating co-designers is one way to make the interaction a little more balanced by acknowledging that institutions and researchers often benefit more than communities in research and design endeavors (40). Compensation, which can take many forms (such as shared meals, household resources, or monetary payment) can go a long way in showing appreciation for community involvement and encouraging future participation (41).

In conclusion, the range of stories produced, the iterative and flexible nature of the co-design process, and the experience of the co-designers demonstrate the effectiveness of leveraging community co-design to create products that have the potential to have a greater impact on outreach programs. Just as important, co-design can foster authentic and sustainable community-academic partnerships, optimizing the use of co-created materials. Indeed, this iterative co-design process, particularly when used within the context of a community-academic partnership, is not limited to outreach stories but can be applied to any situation where science or health content experts need to effectively communicate with a community outside their own healthcare or academic realm. Community co-designer input helps ensure that the content is positively received and understood. Various other materials, including brochures, marketing materials, surveys, focus group questions, and community-friendly reports, can benefit from co-design.

## Data Availability

The raw data supporting the conclusions of this article will be made available by the authors, without undue reservation.
