# Supplementary material for: Graphic-style stories to engage limited resource communities and promote health: methods for iterative co-design with community representatives
Source: Front Public Health. 2025 Sep 8;13:1500711. doi: 10.3389/fpubh.2025.1500711 (PMC12450922; doi:10.3389/fpubh.2025.1500711)
Supplement: Supplementary file 1 [file Data_Sheet_1.pdf]

Supplementary Table 1

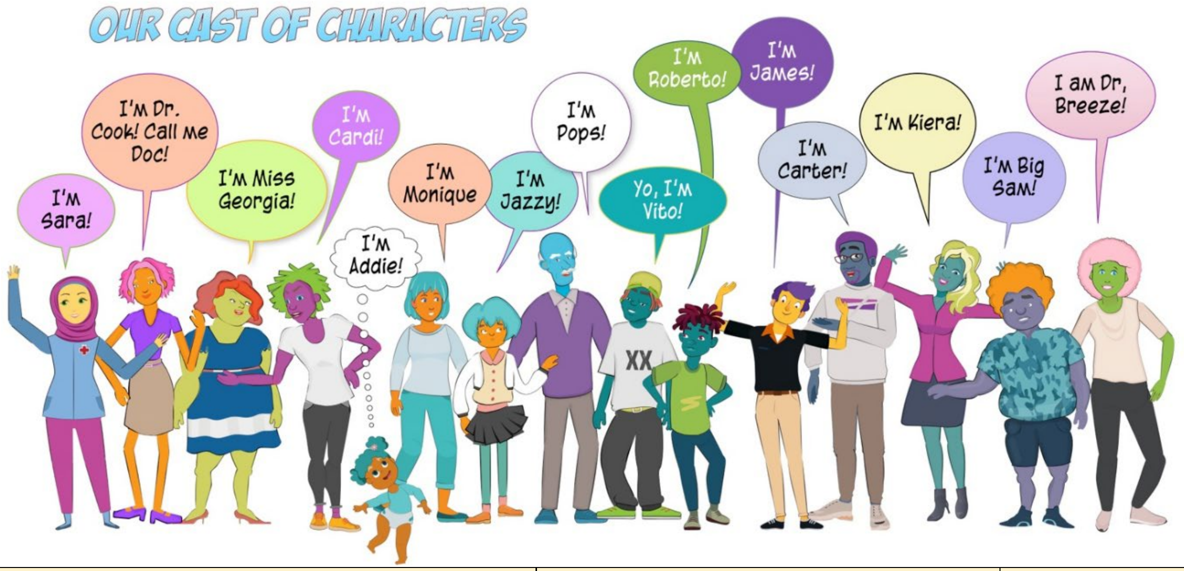

Name	Age	Job	Personality/Details	Health	Back Story
**Science and Health Resource People**					
Carter	50's	University faculty member who does research in environmental health	Outgoing and enjoys being with community members. Involved in citizen science research on the topic of heat islands.	No personal health issues. Has a sister who is a cancer survivor.	Carter is passionate about getting community members involved in his heat island research project. He also lends a hand in other citizen science research projects the community works on, especially in the area of working with data.
Dr. Breeze	40's	Physician/asthma researcher	Jazzy's asthma doctor, also involved in asthma clinical research.	No personal health issues.	Our second physician is an asthma specialist. We meet her when Jazzy has an appointment. Later, she and Jazzy have a telemedicine appointment during COVID-19 lockdown. She has shared knowledge about research protections for human subjects.
Pops	70's	Retired science teacher	Enjoys teaching people about science and helping them figure things out. Sometimes talks too much. Is the school's assistant cross country coach.	Has rheumatoid arthritis. Likes to cook healthy food. Has squamous cell skin cancer and was successfully treated.	Pops is almost everywhere helping other community members get excited about and understanding health and science topics! In stories, he's one of the characters who can be a technical information source because he is a retired science teacher.
Dr. Cook	40's	Physician	Sam's primary care doctor, also involved in citizen science research on cancer.	No personal health issues.	Dr. Cook is a caring person who wants to support the community beyond treating individual patients. She is one of the characters who can be a technical information source because she is a physician.
Kiera	30's	Trauma counselor	Very bubbly and outgoing. Works with Cardi at the community center.	Family history of breast cancer. Is newly pregnant in one of the FAQs on vaccination. Niece Isabel has recurring fevers. (Worry about leukemia)	Kiera has room to grow in our stories because she has only been featured in one story where she and Cardi did a citizen science research project. She has recently been given a family history of breast cancer in our character plan, so she will show up more in that role.

Name	Age	Job	Personality/Details	Health	Back Story
Cardi	30's	Registered dietician and yoga/aerobics instructor at community center	Outgoing and likes to have fun. Encourages community members to come to her classes. Cardi has good intentions, but she can be bossy about health and might take it too far.	No personal health issues. Brother is recovering from a heart attack. Family history of diabetes. Uncle had prostate cancer and was successfully treated.	Cardi has been an aerobics and yoga instructor from the beginning of the stories and has always been involved in supporting other community members' health goals. Now, she is also a Registered Dietician, so she has new ways to support community health.
Sara	20's	Nursing Student		Lump in throat made her concerned about thyroid cancer.	
**Other Adult Community Members**					
Big Sam	40's	Security guard at school	Uses jokes to cover feelings of worry about health. Rents apartment over Miss Georgia.	Has high blood pressure and is overweight. Is concerned about diabetes risk. Older brother died of pancreatic cancer.	Sam works at the same school that Jazzy and Vito attend, so he knows them well. He is also active at the community center.
Miss Georgia	60's	Retired, volunteers at community center	Still hip and having fun. Dances and sings while cooking. Known for her baking. Real sweet lady, kids are grown but no grandkids. Loves neighborhood kids.	Recently diagnosed with diabetes.	Georgia is a real fixture in the community! She's someone who is upbeat and can also give thoughtful advice. She has been active in all the community activities and is especially close to Jazzy.
Monique (Jazzy's mom)	40's	None identified	Always supporting Jazzy to help her with managing asthma.	Breast cancer survivor.	Starting as a minor character without a name besides "Jazzy's mom," she now has the name Monique! She has recently been given the role of breast cancer survivor in our character plan.
**Teen and Younger Community Members**					
James	Older teen	High School Student	Cross-country runner, comic book fan, friends with Vito	On the autism spectrum	
Vito	Older teen	High school student	Acts cool but is truly kind and thoughtful. Likes to joke around. Always looking for snacks. Likes to skateboard. Is a new cross-country runner.	No personal health issues. Dislikes the heat. Vito's father has lung cancer. Vito's uncle has a lingering cough. (Worry about lung cancer.)	Vito may be a bit of a joker, but he also cares, and even organized his own citizen science project about heat islands in the community. Vito and Jazzy go to the same school and hang out at the community center and outdoors in their neighborhood. They have participated in citizen science projects together.
Jazzy	Younger teen	High school student	Sassy personality. Does gymnastics and cheerleading. "Knows it all" even when she doesn't. Caring, likes to help older people.	Has asthma. Has been a research participant and has led a citizen science project around asthma	Everyone loves Jazzy! She's good friends with Vito and Miss Georgia and is involved in her own activities as well as the community. She organized her own citizen science research project with the help of Dr. Breeze. She especially helps and encourages Vito.
Addie	Baby		Jazzy's younger sister	Has eczema.	
Roberto	Preteen	Middle school student	Vito's cousin	Previously treated for leukemia and depression.	
